# Metabolic Rewiring in the Tumor Microenvironment to Support Immunotherapy: A Focus on Neutrophils, Polymorphonuclear Myeloid-Derived Suppressor Cells and Natural Killer Cells

**DOI:** 10.3390/vaccines9101178

**Published:** 2021-10-14

**Authors:** Andrea De Lerma Barbaro, Maria Teresa Palano, Martina Cucchiara, Matteo Gallazzi, Lorenzo Mortara, Antonino Bruno

**Affiliations:** 1 Laboratory of Comparative Physiopathology, Department of Biotechnology and Life Sciences, University of Insubria, 21100 Varese, Italy; 2 Laboratory of Innate Immunity, Unit of Molecular Pathology, Biochemistry and Immunology, IRCCS MultiMedica, 20100 Milan, Italy; mariateresa.palano@multimedica.it (M.T.P.); martina.cucchiara@multimedica.it (M.C.); 3 Laboratory of Immunology and General Pathology, Department of Biotechnology and Life Sciences, University of Insubria, 21100 Varese, Italy; matteo.gallazzi@uninsubria.it (M.G.); lorenzo.mortara@uninsubria.it (L.M.)

**Keywords:** neutrophils, PMN-MDSCs, natural killer cells, tumor metabolism, tumor microenvironment

## Abstract

Leukocytes often undergo rapid changes in cell phenotype, for example, from a resting to an activated state, which places significant metabolic demands on the cell. These rapid changes in metabolic demand need to be tightly regulated to support immune cell effector functions during the initiation and downregulation of an immune response. Prospects for implementing cancer immunotherapy also rest on the idea of optimizing the metabolic profile of immune cell effectors. Here, we examine this issue by focusing on neutrophils and NK cells as cells of increasing interest in cancer immunology and tumor immunometabolism, because they can be targeted or, in the case of NK, used as effectors in immunotherapy. In addition, neutrophils and NK cells have been shown to functionally interact. In the case of neutrophils, we also extended our interest to polymorphonuclear MDSC (PMN-MDSCs), since the granulocytic subset of MDSCs share many phenotypes and are functionally similar to pro-tumor neutrophils. Finally, we reviewed relevant strategies to target tumor metabolism, focusing on neutrophils and NK cells.

## 1. Introduction

“Reprogramming of energy metabolism” and “evading immune destruction” were acknowledged as new cancer hallmarks [1]. Since then, the functional interconnections between the metabolic reprogramming of cancer cells and the metabolic profile exhibited by other cells present in the tumor microenvironment (TME), including cells of the immune system, have been increasingly appreciated [2,3].

Here, we aim to review this fascinating and complex area of investigation by focusing on neutrophils, polymorphonuclear myeloid-derived suppressor cells (PMN-MDSCs) and natural killer (NK) cells. Neutrophils and NK cells can play important and often contrasting roles in the neoplastic process; indeed, these cells, because of their high plasticity, can have both anti-tumor functions and pro-tumor abilities, whereas PMN-MDSCs, i.e., immature neutrophils with similarities to neutrophils, are able to exert strong inhibitory effects on immune responses, as well as several pro-tumor and pro-angiogenic functions. Moreover, the impact of the metabolic rewiring in the TME and the subsequent readout on neutrophils and NK cells is still less exhaustively investigated, compared to macrophages and T lymphocytes [4,5,6]. In addition, neutrophils and NK cells have been reported to interact, generating contrasting effects in relation to the TME [6,7,8,9,10].

Throughout the review, we direct attention towards glucose and lipid metabolism, while not addressing amino acid metabolism. Before going into the topic, we will briefly introduce the role of neutrophils, PMN-MDSCs and NK cells in health and in the neoplastic process.

## 2. Neutrophils and Polymorphonuclear MDSCs in Health and in Cancer

Neutrophils are myeloid polymorphonuclear leukocytes, involved as the first line of defense during inflammation and infections, particularly against bacterial microorganisms. Neutrophil main effector functions include phagocytosis, reactive oxygen species ROS production and degranulation [5]. Neutrophil extracellular traps (NETs) constitute a recently described form of the antimicrobial weapons of neutrophils. NETs are fibers of chromatin released from neutrophils in an active process named NETosis [11]. Neutrophils account for 40% to 70% blood nucleated cells in humans and 10–25% in mice. Moreover, neutrophils in bloodstream are short-lived cells that require a constant turnover from bone marrow precursors [5]. Early studies suggested that neutrophils were dull, uninteresting cells mostly involved in acute inflammation. Studies in recent years have highlighted an unexpected plasticity of neutrophils and their involvement in chronic inflammation, in the interface between innate and acquired immunity and in cancer [5]. Indeed, neutrophils engage in complex bidirectional interactions with lymphoid cells and macrophages, mostly through their ability to acquire peculiar phenotypes and ability to release different arrays of cytokines [5].

The relevance of neutrophils in cancer has been extensively recognized [4,5]. As myeloid cells, neutrophils can exert dual and opposite functions in tumor progression, both in humans and mice [12,13,14,15]. An increase in neutrophils in blood usually accounts for a sign of poor prognosis. Mimicking the nomenclature used to define macrophages (M1-like vs. M2-like/TAM), tumor-associated neutrophils (TANs) can acquire at least two different phenotypes, associated with opposite biological behaviors: N1 neutrophils, endowed with anti-tumor activities, and N2 neutrophils, which support tumor progression as a consequence of their immunosuppressive and pro-angiogenic properties. Recent studies pointed out that the definition of N1 and N2 does not completely parallel Th1/Th2 or M1/M2 dichotomies because, in contrast to these cells, IFNγ, IL-12 and IL-4 are not key cytokine drivers of the functional polarization of neutrophils [5,12,13,14,15].

Pro-tumor activities by TANs can cover a wide array of mechanisms [4,5]. TANs can secrete matrix metalloproteinase-9 (MMP-9) that in turn supports the release of vascular endothelial growth factor (VEGF) from the extracellular matrix (ECM) to promote angiogenesis. TANs can secrete arginase 1, which inhibits CD8 T cells, generating an immunosuppressive environment. TANs also produce reactive oxygen species (ROS) that induce damage DNA, resulting in genotoxic effects on tumor cells. Conversely, TANs can also mediate anti-tumor responses by the direct killing of tumor cells and by participating in cellular networks that mediate anti-tumor resistance [16,17,18].

As anticipated, there is still an active debate concerning the relationship between neutrophils and, in particular, pro-tumor N2 and PMN-MDSCs [5,19]. Indeed, N2 neutrophils and PMN-MDSCs share origin and several phenotypic features, and both display pro-tumor functions. MDSCs are defined as a heterogeneous population of mostly immature myeloid cells endowed with immunosuppressive activity. MDSCs include two different subpopulations: PMN-MDSCs [20] and monocytic MDSCs (M-MDSCs). Both N2 and PMN-MDSCs display a pro-tumor phenotype [21]. While in healthy individuals, PMN-MDSCs are almost undetectable, in several pathological conditions such as cancer, chronic infectious disease, sepsis, trauma and others, these cells accumulate, as well as M-MDSCs, and coexist with neutrophils [19]. In humans as well as in murine models, PMN-MDSCs represent the main cell subset of MDSCs [19]. Human PMN-MDSCs are defined as CD14^−^CD11b^+^CD15^+^ cells or CD14^−^CD11b^+^CD66b^+^ cells, whereas in murine cells as CD11b^+^Ly6G^+^Ly6C^lo^ [21,22,23]. Despite high phenotypic similarity between human neutrophils and MDSCs, recently, it has been shown that the lectin-type oxidized LDL receptor 1 (LOX-1) might represent a specific marker of human PMN-MDSCs [24]. However, further studies seem necessary to confirm this thesis [21]. Moreover, very recently, using single-cell RNA-seq, cell mass spectrometry, flow cytometry and functional analysis, in tumor-bearing mice, Veglia et al. described three distinct subsets of neutrophils, i.e., classical neutrophils, PMN-MDSCs and activated PMN-MDSCs, based on the expression of CD14 [22]. These two latter cells are endowed with potent immune suppressive activity. Interestingly, activated PMN-MDSCs are present only in the tumor mass, and similarly, in human cancer patients, two distinct peripheral blood populations of neutrophils have been characterized: classical PMNs and PMN-MDSCs. Of note, a gene signature analysis indicated that tumor PMN-MDSCs had high similarities with mouse activated PMN-MDSCs and correlated with negative clinical outcome [21], paving the way for potential selective targeting approaches.

## 3. NK Cells in Health and in Cancer

NK cells, large granular lymphocytes from innate immunity, are directly involved in the elimination of virus- and bacteria-infected cells and in the immunosurveillance against malignant transformed cells [25]. Recently, NK cells have been included in the family of innate lymphoid cells [26]. NK cells can discriminate their target cells from healthy cells based on an aberrant expression of MHC class-I molecules and cell stress markers [27,28]. Low MHC class-I expression is kept under surveillance by a host of inhibitory receptors, whereas de novo expression of endogenous stress signals is surveyed by a plethora of activating receptors. Moreover, NK cells are strong mediators of antibody-dependent cellular cytotoxicity (ADCC) [27,28].

NK cells account for 5% to 15% of the mononuclear cells in the blood. On the basis of the surface antigen expression, two major NK cell subsets can be distinguished: CD56^dim^CD16^+^ NKs (90–95% of total circulating NKs), able to produce perforin and granzymes and that mediate the ADCC; and CD56^bright^CD16^-^ NKs (5–10% of total circulating NKs), able to release anti-tumor Th1 cytokines, such as IFNγ and TNFα [25,26]. A third NK cell subset has been characterized in the developing decidua, during pregnancy [29,30]. This peculiar NK cell subset, defined as decidual NK (dNKs), is necessary to generate the tolerogenic hosting environment for the developing fetus and supports the formation of the spiral artery that supplies oxygen and nutrients [29,30]. NK cell functions go beyond those of killers, and they are now appreciated and investigated also for their immunoregulatory roles. In this scenario, NK cells exert important functions in innate but also acquired immunity, by establishing complex bidirectional interactions with other immune cells both from innate and adaptive immunity [6,8,31,32].

In cancer patients, NK cells have been found to be altered in their anti-tumor functions, as a consequence of several TME-related factors (TGFβ, adenosine, PGE_2_), TME chemical conditions (acidity, abundance of ROS and lactate, hypoxia) and interactions with MDSCs, and in particular with PMN-MDSCs [6,33,34,35,36,37]. Major mechanisms generating anergic NK cells include the decrease in the expression of the activation receptor NKG2D, together with cell exhaustion (by increased expression of PD-1, Tigit, CD96, TIM3) and impaired degranulation capabilities and release of IFNγ [38,39,40,41,42]. Recently, decidual-like pro-angiogenic NK cells have been identified in non-small cell lung [43], colorectal [44] and prostate cancers [45] and in the pleural effusions of patients with metastatic cancers [46]. These decidual-like NKs acquire the CD56^bright^CD9^+^CD49a^+^ decidual-like phenotype [43,45,46,47,48], and support tumor angiogenesis in a TGFβ [43,45] and STAT3-dependent [47] manner, by favoring angiogenesis, directly acting on endothelial cells or by generating pro-angiogenic M2-like/TAMs [45].

Within the era of immunotherapy, the relevance of NK cells as effectors for cell therapy has been increasingly recognized, both by researchers and clinicians [6,49,50]. Compelling evidence indicates that NK cells contribute to cancer immunosurveillance, possibly in the early phases of tumorigenesis [51]. Moreover, some tumors, especially those of hematopoietic origin, are targets of NK cytotoxic activity. Nevertheless, in clinically established solid tumors, NK cells are often poorly represented and frequently display phenotypic and functional features reminiscent of dNK [6,43,45,46,47,48]. Based on this knowledge and on some failures in the use of chimeric antigen receptor (CAR) T cells, NK cells are now being explored as a new tool for immunotherapeutic approaches [6,41,49,50].

## 4. Metabolism and Cancer, a Focus on Neutrophils and NK Cells

The notion that cancer cells harbor a different metabolic profile with respect to healthy cells was introduced almost 100 years ago [52,53]. In the presence of oxygen, normal cells are expected to process glucose, first to pyruvate (via glycolysis) within cytosol and thereafter to carbon dioxide (via the tricarboxylic acid, TCA, cycle and oxidative phosphorylation OXPHOS) in mitochondria [54]. Conversely, cancer cells are able to maintain a high rate of glycolysis, even in the presence of oxygen, a phenomenon known as ‘aerobic glycolysis’ or the ‘Warburg effect’ [54]. Accordingly, cancer cells convert much of the pyruvate into lactate, which is then released into the extracellular compartment. As a matter of fact, at first the metabolic profile of cancer cells has been perceived as paradoxical, since the metabolic efficiency of glycolysis is much lower than that of oxidative phosphorylation (2 ATP moles versus 36 ATP moles per glucose mole). Consequently, cancer cells consume high amounts of glucose and significant areas in tumors exhibit lactic acidosis. It is well established that a cell with a high proliferative rate needs efficient ATP production, abundant production of building blocks and macromolecules and a tight maintenance of the redox balance. Nowadays, the alterations in cancer metabolism are explained, since anaerobic glycolysis meets these requirements, being largely anabolic. In a rapidly proliferating tumor cell, diversion pathways of glycolysis, such as the pentose phosphate pathway (PPP), are essential for generating important biomolecules such as NADPH and ribose sugars. The NADPH is crucial for cancer cells to fulfill various metabolic requirements, such as ATP production and lipogenesis, as well as to contrast the oxidative stress. Similarly, the ribose sugar, as an integral part of nucleotides, is crucial for fast-dividing cells. As a whole, cancer cells are able to utilize anabolic reactions to generate diverse intermediates for the synthesis of nucleic acids, lipids and proteins. It should be emphasized that in most cases the metabolic profile of the tumor cell is not due to mitochondrial alterations. Indeed, in cancer, mitochondrial defects are rare, and most tumors retain their capacity for oxidative phosphorylation [55]. Moreover, the metabolic reprogramming of cancer is by and large transcriptionally regulated by oncogenes and mutated tumor suppressors [55,56,57].

Over the past fifteen years, it has become increasingly clear that the metabolic portrait of cancer is more complex than initially assumed [55]. Glucose is not the only source of metabolic energy; fatty acids, amino acids and catabolites produced in the TME are also important for tumor growth. Consequently, in addition to glycolysis, several other pathways are involved in the production of metabolic energy and biomolecules required for cell proliferation, including the TCA cycle, coupled to OXPHOS, fatty acid oxidation (FAO) and glutaminolysis. As a matter of fact, the Warburg metabolic phenotype remains the best characterized, but it is not the only one present in cancer cells. As a rule, multiple pathways for energy metabolism coexist in a cell, even if among these generally one is dominant. Furthermore, it was appreciated that a prevalent aerobic glycolysis is present in several cell types characterized by a high proliferation rate, starting with cells of the immune response after activation. Thus, the “altered” metabolic profile of cancer cells reflects the cooption of a normal physiological mechanism. Finally, in the last decade, the importance of interaction/cooperation between cells with different metabolic profiles in the TME has been progressively appreciated [3]. Predominantly glycolytic tumor cells cooperate with mainly OXPHOS-based tumor cells; in addition, tumor cells cooperate with mesenchymal cells, particularly fibroblasts.

### 4.1. Metabolism in Immune Cells

The field of immunometabolism stems from the growing evidence that diverse differentiation or functional states of the same cell of the immune system often have different metabolic signatures. However, it is important to keep in mind that in physiological conditions, within cells, different pathways for energy metabolism coexist, even if there is often one that prevails [58]. For example, whereas naïve and memory T cells utilize OXPHOS to derive ATP for their needs, proliferating T lymphocytes reprogram their metabolism and switch to glycolysis for fulfilling the energetically demanding processes of cell division and effector functions. In detail, while Th1 cells predominantly use aerobic glycolysis and some OXPHOS, T regulatory (Treg) cells preferentially use lipid oxidation. Metabolic rewiring has been thoughtfully investigated even in macrophages [59,60,61,62]. M1-like macrophages preferentially utilize the glycolytic pathway with a broken TCA cycle to generate inflammatory mediators, whereas M2-like/TAM macrophages utilize β-oxidation with an intact TCA cycle [63,64].

The metabolic plasticity of immune cells is primarily due to their mobility, since they are exposed to various environments wherein the availability of nutrients differs [65,66]. Moreover, the adoption of diverse metabolic profiles is not only a strategy to optimize the production of ATP and biosynthetic precursors, but it is often intrinsically linked to, and may even determine, the functional output of that precise cell [67].

Currently, the study of immunometabolism extensively contributes to the understanding of cellular interactions in the TME. Here, we focus on metabolism in neutrophils and NK cells, in health and later in cancer. As compared to T cells and macrophages, metabolism in neutrophils and NK is poorly understood and still represents a challenging topic to be explored.

### 4.2. Neutrophil Metabolism

Early studies suggested that neutrophils mainly use glycolytic metabolism. Recent advances in the immunometabolism field have partly confirmed this conclusion, highlighting, however, a more multifaceted portrait [68]. Mature but resting neutrophils use glycolysis as almost their only source of energy. Following activation, mature neutrophils use, in parallel to glycolysis, different mitochondria-dependent metabolic pathways, not for energy production but to support specialized effector functions, such as ROS generation, NETosis, phagocytosis, chemotaxis and degranulation [11,69].

Activated neutrophils often encounter harsh, ischemic environments where the availability of glucose and oxygen is low; in these cases, neutrophils resort to gluconeogenesis and glycogenesis. Accordingly, neutrophils have been shown to contain glycogen stores within granules [70]. Finally, immature neutrophils precursors in the bone marrow present a metabolism significantly different from mature neutrophils, being based on the beta-oxidation of fatty acids followed by OXPHOS in mitochondria. Consequently, in immature neutrophils there is a higher mitochondrial biomass as compared to mature neutrophils; however, it should be noted that mitochondria in mature neutrophils, although few, are functional [69].

### 4.3. Neutrophil Metabolism in Cancer

Currently, not much is known about the energy metabolism of TANs. In the first place, neutrophils in TME are exposed to by-products of tumor and stroma metabolism; indeed, extracellular acidosis from cancer-generated lactic acid secretion acts as a regulator of neutrophil apoptosis and function. Moreover, several lines of evidence suggest that TME acidity significantly enhances the tumor-promoting functions of TANs [5].

**Figure 1:**
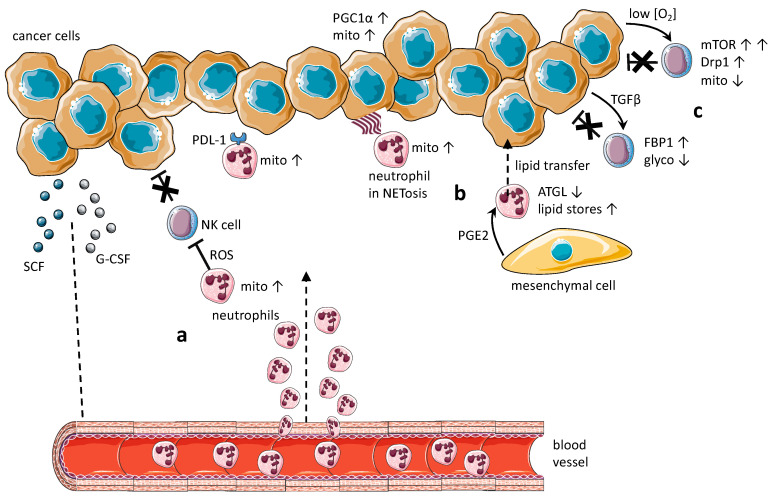
**Metabolic profile in pro-tumor immune cells with a focus on neutrophils and NK cells**. (**a**) Cancer cells secrete growth factors and cytokines, such as SCF and G-CSF [71,72], that recruit immature, pro-tumor neutrophils in the TME. These neutrophils rely mostly on a mitochondrial metabolism and are immunosuppressive for T and potentially for NK cells [71], promote cancer metastasis through NETosis [72] and express increased levels of PD-L1, contributing to the immunosuppressive milieu in the TME [73]. (**b**) Pro-tumor neutrophils may support metabolic competence in cancer cells. Two examples: neutrophil elastase released from NETs activates TLR4 signaling in cancer cells, leading to upregulation of PGC1a, the master regulator of mitochondria biogenesis [74]; upon interaction with resident mesenchymal cells, pro-tumor neutrophils are induced to accumulate lipids due to inhibition of the ATGL enzyme. Thereafter, lipids stored in neutrophils are transported to tumor cells, increasing metabolism in these cells [75]. (**c**) Molecular cues from the TME lead to metabolic and functional changes in NK cells. Early glycolytic, cytotoxic, and anti-tumor NK cells become dysfunctional in later phases of tumorigenesis due to the over-expression of FBP-1 and concomitant inhibition of glycolysis. This process is possibly driven by TGFβ [76]. Hyperactivation of mTOR signaling in cytotoxic anti-tumor NK cells, due to the persistent stress of the hypoxic TME, leads to activation of Drp1 and impaired mitochondrial metabolism. As a result, NK cells become dysfunctional [77]. The described mechanisms refer to cancers in the mammary gland [71,72], in the lung [76], in the colon [74], in the liver and in the pancreas [73].

Another important aspect is the mobilization of neutrophils from hemopoietic sites in bone marrow and recruitment in the tumor mass, where neutrophils are endowed with pro-tumor metabolic signatures. This topic has been recently investigated in three articles and is illustrated in Figure 1. 

In bone marrow, immature neutrophils express c-Kit receptor, whose ligand is the stem cell factor (SCF) [71]. In a murine model of mammary carcinomas (4T1), tumor cells produce SCF, selecting for, in a glucose-poor microenvironment, immature neutrophils characterized by mitochondrial metabolism. In these neutrophils, ROS production is dependent on fatty acid beta-oxidation, followed by OXPHOS. ROS, in turn, is immunosuppressive for T cell effective responses and, although not analyzed in the article, probably for NK cells [72].

In another study performed in the murine 4T1 mammary carcinoma model, tumor derived G-CSF has been reported to mobilize immature low-density neutrophils (iLDNs) that promote breast cancer liver metastasis, mostly through NETosis. iLDNs can perform pro-metastatic functions under metabolically challenging conditions, such as low glucose, due to their mitochondria-dependent, enhanced global bioenergetic capacity [73].

Extracellular nucleotides, mostly adenosine and ATP, are major molecular constituents of the TME. By activating the purinergic P1 or P2 receptor, extracellular nucleotides exert a contrasting effect on tumor progression, the promotion of tumor cell proliferation and an increased anti-tumor activity of immune cells [74]. In both humans and mice, a subset of neutrophils lacking expression of the purinergic P2RX1 receptor is mobilized from bone marrow and recruited in pancreatic cancer liver metastases. RNA sequencing studies show that tumor-infiltrating P2RX1-deficient neutrophils express increased levels of immunosuppressive molecules, including PD-L1, and have enhanced mitochondrial metabolism. This work is also interesting because it suggests that PD-L1 expression is linked, at least in the neutrophil, with metabolic reprogramming [75].

Taken together, these studies account for pro-tumoral neutrophils in the TME endowed with mitochondrial metabolism, frequently a feature of immature granulocytes (Figure 1a,b). Immature neutrophils are placed in the bone marrow but, upon massive inflammatory processes and emergency granulopoiesis, they move to peripheral tissues [5]. Therefore, the TME recruits, and hence selects for, pro-tumoral neutrophils. The alternative hypothesis is that the TME induces differentiation in neutrophils already present in the tumor mass from an anti-tumoral N1 phenotype to a pro-tumoral N2 phenotype [5]. While both scenarios are possible, the current evidence mostly supports the former.

### 4.4. NK Cell Metabolism

Mature resting NK cells use OXPHOS to supply their homeostatic requirements [76,77]. In response to cytokines such as IL-2, IL-12, IL-15, and IL-18, activated NK cells increase both glycolysis and OXPHOS pathways to fuel biosynthetic precursors, necessary for proliferation and effector functions [78]. Indeed, OXPHOS pathway inhibition in NK cells correlates with a significant decrease in IFNγ secretion and cytotoxic activities [78].

In the developmental process of NK cells, as far as during the process of education, a shift towards a more glycolytic metabolic profile is observed. Several studies have yet to comprehensively measure the metabolic activity of NK cells at different stages of development [78]. Recently, it was elucidated that metabolic rewiring in NK cells activated by IL-12, IL-15 and IL-18 depends on three master regulators: TORC1, a signal transducer, SREBP and cMYC acting as transcriptional factors [76,79,80,81]. The mTOR/TORC1 complex, located in the cytosol, is an important sensor that integrates signals of nutrient availability, activation, and growth. Rapamycin, a selective inhibitor of mTORC1, effectively blocks changes in NK cells’ metabolism.

Finally, NK cells exhibit distinct metabolic signatures depending on whether these cells are immature or mature. Mature cells may themselves be in a resting, activated or memory state. Activated effector NK cells can be cytotoxic or regulatory. An important type of pro-angiogenic regulatory NK cell that works in a hypoxic environment is represented by the uterine/decidual NK (dNK) cells. It is believed that dNK cells have a metabolic profile distinct from cytotoxic NK cells, but little evidence is currently available on this very intriguing topic [82].

### 4.5. NK Cell Metabolism in Cancer

Metabolic reprogramming of NK cells is a requisite to display enhanced effector functions, but this process could be impaired under the immunosuppressive conditions characterizing the TME [78]. The TME can impair NK cell activity by acting on the metabolic profile of these cells, based on three principal routes: (a) TGFβ, a relevant immunosuppressive cytokine largely present in the tumor micro (tissue-local) and macro (systemic) environments, able to inhibit metabolic activity in NK cells; (b) metabolites (lactic acid, adenosine and kynurenine, a catabolite of tryptophan) produced in the TME by tumor and stromal cells that in turn can inhibit metabolic activity in NK cells; and (c) nutrients/substrates and oxygen, crucial for the maintenance of NK cell activity and that are present in the TME in limiting amounts [33].

Four recent articles investigating the metabolic profile of cancer-infiltrating or cancer-associated NK cells are discussed below and illustrated in Figure 1.

In a KRAS-driven mouse model of lung cancer, tumor-infiltrating NK cells over-express fructose-1,6- biphosphatase 1 (FBP-1) [83]. FBP-1 is the rate-limiting enzyme for gluconeogenesis (the synthesis of glucose from catabolites and noncarbohydrate carbon sources) and simultaneously inhibits glycolysis. As a result of this switch in energy metabolism, NK cells become dysfunctional. This process is possibly driven by TGFβ, and is interestingly gradual, with NK cells being anti-tumor in the initiation phase and then becoming progressively anergic with the subsequent promotion and progression phases.

PGC-1a is a transcriptional coactivator that drives the transcription of genes critical for mitochondrial function, and its function mostly lies in response to energetic stress. A conditional knockout model of PGC-1a in NK cells shows the critical role of this transcriptional coactivator in maintaining mitochondrial function in NK cells during an immune response against cancer. The NK cells of PGC-1a-deficient mice fail to clear B16F10 tumors to the level of WT cells [84].

Tumor-infiltrating NK cells in human liver cancers have small, fragmented mitochondria and diminished mitochondrial health, whereas liver NK cells outside tumors, as well as peripheral NK cells, have normal large, tubular mitochondria [85]. This fragmentation correlates with reduced NK cytotoxicity and NK cell loss, resulting in tumor evasion of NK cell-mediated surveillance.

Whereas optimum mTOR activity is pivotal for the function of NK cells, continuous hyperactivation of mTOR signaling, due to the persistent stress of the hypoxic TME, drives exhaustion and reduced anti-tumor capacity in NK cells. Hypoxia leads to activation by phosphorylation of Drp1, the main regulator of mitochondrial fission [86].

Circulating NK cells from patients with HCV-related hepatocellular carcinoma were studied in comparison to NK from healthy controls by transcriptomic, phenotypic and functional analyses [87]. The results of this unbiased approach showed differential regulation in pathways regarding energy metabolism, cell motility/adhesion and reduced cytokine production and degranulation, despite a prevalent phenotype of terminally differentiated NK cells. High SMAD2 phosphorylation in NK cells suggested TGFβ as a possible mediator of this dysfunction [87].

Altogether, these studies suggest that both glycolysis and oxidative phosphorylation, and thus mitochondria, are critical for the anti-tumor activity of TME-infiltrating NK cells, which is consistent with what is known about the general metabolic profile of mature/educated and activated NK cells (Figure 1c). The somewhat surprising fact, however, is that there do not seem to be any studies showing that catabolites produced in the TME redirect NK cells from anti-tumor to pro-tumor activity. Indeed, in general, the observed effect is an inhibition of NK cell function. In analogy to neutrophils, this evidence could be explained by the fact that the TME induces anergy and/or apoptosis in NK cells with anti-tumor metabolic profiles, while simultaneously recruiting pro-tumor NK cells.

There is still a gap of knowledge to be filled in regarding the metabolic profile of regulatory, pro-angiogenic NK cells that are physiologically present in the decidua [29,30] and have a pathological counterpart in some solid cancers [6,43,45,46,47,48,88].

### 4.6. Metabolic Cooperation in the TME between Neutrophils and Cancer Cells

The ability of tumors to modify/modulate the microenvironment is a key concept to understand cancer metabolism. Among cancer cells, some maintain a glycolytic phenotype, while others predominantly use the TCA cycle coupled with OXPHOS. The lactate abundantly released by glycolytic cancer cells located in the hypoxic area in the tumor mass feeds cancer cells located in vascularized areas. What is more, interactions between cancer cells and other cells in the TME allow metabolites to be shifted from stromal cells, mostly fibroblasts, to meet metabolic demands and maintain ATP production in cancer cells. Indeed, this is the reverse Warburg effect, when glycolysis in the cancer-associated stroma metabolically supports adjacent cancer cells [3]. These cell interactions are understood as a metabolic symbiosis and even “corrupted” immune cells could take part in these cell collaborations. What about neutrophils or NK cells? Recently, two reports were published about the support of neutrophils in the energy metabolism of cancer cells.

In a murine model of colon carcinoma, the growth of subcutaneous tumor implants and hepatic metastasis slowdown in PAD4-KO mice, genetically not able to undergo NETosis. Tumors in PAD4-KO mice exhibit decreased proliferation, increased apoptosis and augmented oxidative stress. Moreover, PAD4-KO cancer cells are characterized by decreased mitochondrial density biogenesis and function. Mechanistically, neutrophil elastase released from NETs activates TLR4 in cancer cells, leading to upregulation of PGC1a, the master regulator of mitochondria biogenesis. In conclusion, NETs can directly alter the metabolic programming of cancer cells, resulting in increased tumor growth [89]. In the 4T1 breast cancer model, neutrophils placed in the pre-metastatic lungs are induced to accumulate lipids upon interaction with resident mesenchymal cells. Lung mesenchymal cells elicit this process through repressing the adipose triglyceride lipase (ATGL) activity in neutrophils in PGE_2_-dependent and -independent manners. In turn, lipids stored in lung neutrophils are transported to metastatic tumor cells through a macropinocytosis–lysosome pathway, endowing tumor cells with augmented survival and proliferative capacities. Neutrophils serve as an energy reservoir to fuel breast cancer lung metastasis [90].

From the studies discussed in Section 4.3, Section 4.5 and Section 4.6, it is possible to draw some general conclusions regarding how neutrophils or NK cells may exert pro-tumor functions in the TME based on metabolic reprogramming (Figure 1a). Cancer cells secrete growth factors and cytokines that recruit immature, pro-tumor neutrophils in the TME. These neutrophils rely mostly on mitochondrial metabolism and promote tumor progression through a variety of mechanisms (Figure 1b), and pro-tumor neutrophils may cooperate with cancer cells, supporting their metabolic competence (Figure 1c). Molecular cues from the TME lead to metabolic changes in NK cells, with a consequent loss of anti-tumor cytotoxic activity.

### 4.7. Do NK Cells Exert an Editing Function on Cancer Cell Metabolism through Cytotoxic Activity?

Some evidence suggests that immune cells may be involved in shaping the metabolic features of cancer cells through an editing process. This may be the case for NK cells that recognize and kill a broad category of cancer cells, characterized by low MHC-I expression and high surface display of activating receptor ligands. Indeed, in cancer cells, the cell metabolism landscape is linked to the level of MHC class-I expression. The cellular activity of the MAPK ERK5 drives both respiration (OXPHOS) and high MHC class-I expression; conversely, fermentation (glycolysis) and low ERK5 are associated with low MHC-I [91]. In this way, a link can be envisaged between cancer immunosurveillance and the tumor cell metabolism. According to the reverse Warburg hypothesis, in the tumor bed both glycolytic and OXPHOS-driven cancer cell areas coexist [3].

Anti-tumor, actively cytotoxic NK cells could shift the balance toward cancer cells, depending on OXPHOS metabolism. Whereas direct evidence supporting this view is currently lacking in in vivo studies, the functional outcome in cancer cell lines on the effector function of NK cells and cytotoxic T lymphocytes (CTLs) of MHC-I modulations, due to changes in tumor cell metabolism, has been reported [92].

### 4.8. Crosstalk between Neutrophils or PMN-MDSCs and NK Cells and Cancer Metabolism

To our knowledge, no article has been published on neutrophil and NK cell crosstalk in relation to cancer metabolism. Nevertheless, metabolic interactions in cancer between PMN-MDSCs or whole MDSCs and NK cells have been investigated in murine models in at least two reports. MDSCs are a heterogeneous population of pathologically activated myeloid precursors and relatively immature myeloid cells that accumulate under many pathological conditions. In both mice and humans, PMN-MDSCs represent the most abundant population of MDSCs [19]. Lactate dehydrogenase-A (LDH-A), the enzyme responsible for conversion of pyruvate to lactate, is often highly expressed in tumor cells. In mice the injection of an LDH-A KO pancreatic cancer cell line gives rise to smaller tumors than in mice injected with parental cells [93]. This decreased growth is accompanied by lower frequency, the reduced immunosuppressive capability of MDSCs in the spleen and the improved cytotoxic function of splenic NK cells. Further ex vivo experiments suggest that tumor-derived lactate restrains NK cell function via direct inhibition of cytolytic function, as well as indirectly by increasing the numbers of MDSCs that, in turn, suppress NK cell cytotoxicity [93].

The tissue stress generated by major cancer surgery activates granulocytic MDSCs [94], which in turn induce an over-expression of scavenger receptors on NK cells present in the tissue. Therefore, these NK cells internalize lipid particles, reprogram their metabolism and become dysfunctional by losing tumoricidal activity [95]. This sequence of events predisposes cells to metastatic growth. The molecular mechanisms of how MDSCs regulate scavenger receptor expression and lipid accumulation in NK cells remain unclear.

Interestingly, this line of research is consistent with the recent discovery that in a lipid-rich environment, and hence in obesity, NK cells accumulate lipids, reprogramming their metabolism. These lipid-laden NK cells display decreased effector functions and fail to eradicate tumor growth in vivo [96], as further discussed below.

## 5. Targeting Tumor Metabolism: A Focus on Neutrophils and NK Cells

The last ten years have witnessed the success of new cancer immunotherapy strategies, in particular, checkpoint inhibitors and CARs [97]. A relevant and a persisting limitation remains that these therapeutic approaches, in particular, checkpoint inhibitors, are successfully effective in a small percentage of patients. While it is essential to develop markers to identify patients who will benefit from immunotherapy, another potential solution to overcome this problem is to use combination therapy strategies. In this scenario, there is a strong interest in strategies aimed at combining the use of checkpoint inhibitors or CARs with treatments that improve the metabolic profile, and hence effectiveness, of the cellular effectors of the immune response [98].

### 5.1. Boosting Metabolism in Anti-Cancer NK Cells

**Figure 2:**
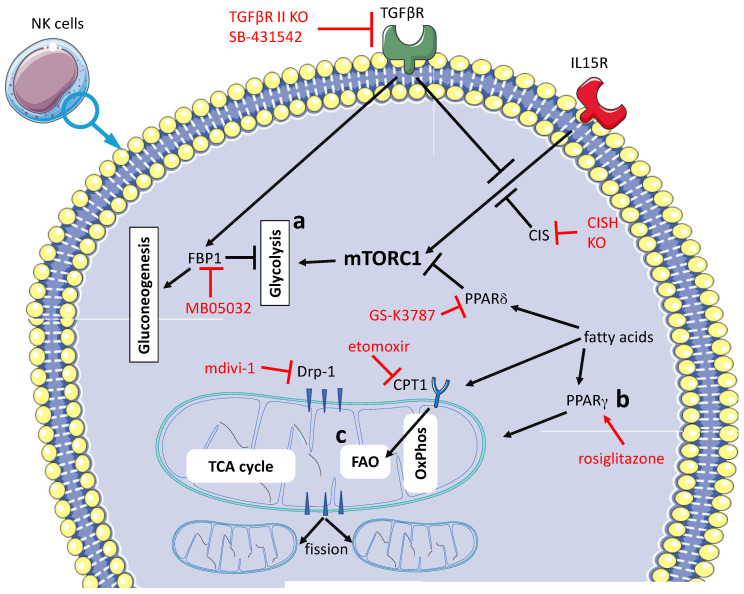
**Pharmacological treatments or transgenesis aimed at restoring in the TME the anti-tumor activity of NK cells’ three main targets**. (**a**) Fostering glycolysis can be pursued by several approaches: inhibition of TGFβR signal transduction [96,97], over-activation of IL-15R signal transduction [98,99], inhibition of gluconeogenesis [76] and inhibition of PPARδ [93]. In most cases, these interventions produce an activation of mTORC1. (**b**) Rescuing homeostasis of mitochondria through treatment with a PPARγ agonist [100] or inhibition of Drp-1 [77]. (**c**) Inhibiting fatty acid oxidation [93]. Therapeutic interventions are outlined in red.

Several NK cell-based cancer immunotherapy strategies are currently under investigation and optimizing the metabolic profile of these cells could be a profitable way to unlock their full therapeutic potential [78]. To this end, drugs directed against different targets have been investigated in preclinical murine models. In addition, ex vivo genetic modification of NK cells has been attempted for use in adoptive cell transfer (ACT) procedures, as illustrated in Figure 2.

Glycolysis is essential for an efficient effector function of NK cells. The “central hub” in the regulation of glycolysis in NK cells is mTOR, more specifically mTORC1 [99]. Signal transductions downstream of IL-15R and TGFβR are functionally interconnected; in fact, IL-15 promotes glycolysis instead. TGFβ inhibits glycolysis, at least in part because this cytokine interferes with the signaling downstream of IL-15R [36]. The signaling downstream of TGFβR has been inhibited by the drug SB-431542 [100] or by transgenesis [101], and in both cases, an enhancement of the IL-15/mTORC1 axis and of glycolysis has been reported. This event was accompanied by increased effector functions in NK cells and direct or indirect evidence of decreased tumor growth. NK cells engineered with CD28ζ-based CAR-CD19 and IL-15 are further knocked out for the CISH gene, which encodes for cytokine-inducible SH2-containing protein (CIS). CIS is induced following IL-15 stimulation and acts as a negative regulator of IL-15 signal transduction. These events modified NK cells to exhibit an improved metabolic profile and an enhanced in vivo anti-tumor activity following adoptive cell transfer (ACT) [102]. The genetic ablation of the CIS-negative regulator of IL-15 signaling has been applied also in induced pluripotent stem cell-derived NK cells (iPSC-NK cells) [103]. Moreover, signaling downstream of TGFβR may drive, in tumor-infiltrating NK cells, the over-expression of fructose-1,6-bisphosphatase (FBP1). The expression of this key regulator of gluconeogenesis is associated in NK cells with decreased glycolysis and reduced viability. Ex vivo inhibition of FBP-1 with MB05032 restores glycolysis and cytotoxicity in NK cells that can suppress tumor growth when transferred in vivo [83]. Another relevant aspect is that, in a lipid-rich environment, NK cells tend to become anergic and lose anti-tumor activity [78,104]. A recent article has highlighted that this tendency can be counteracted pharmacologically. Indeed, the fatty acid sensor PPARδ inhibits mTORC1 and thus glycolysis, and the PPARδ antagonist GS-K3787 restores in vitro the anti-tumor function in NK cells [96]. Furthermore, the fatty acid translocase inhibitor etomoxir CPT1 suppresses FAO in NK cells, resulting in an OXPHOS to glycolysis metabolic switch. Again, NK cells regain anti-tumor activity in vitro, indirect evidence that they can suppress tumor growth [96]. As discussed previously, along with glycolysis, mitochondrial metabolism also contributes to the effector functions of anti-tumor NK cells. In a lipid-rich environment, the lipid sensor PPARγ agonist rosiglitazone, which is already in use in clinics [105], promotes IFNγ secretion, and thus the in vitro anti-tumor activity of NK cells. This effect is likely due to the enhancement of mitochondrial membrane potential in NK cells. However, this putative therapeutic effect is not accompanied by significant inhibition of tumor growth [106]. Finally, in a hypoxic environment, mitochondrial fragmentation in NK cells infiltrating the cancer tissue is associated with impaired function. Pharmacological inhibition of Drp1, which mediates mitochondria fission, with mdivi-1 or dynasore restores in vivo mitochondria morphology and NK anti-tumor functions [85].

### 5.2. Targeting Neutrophil/MDSC Metabolism

**Figure 3:**
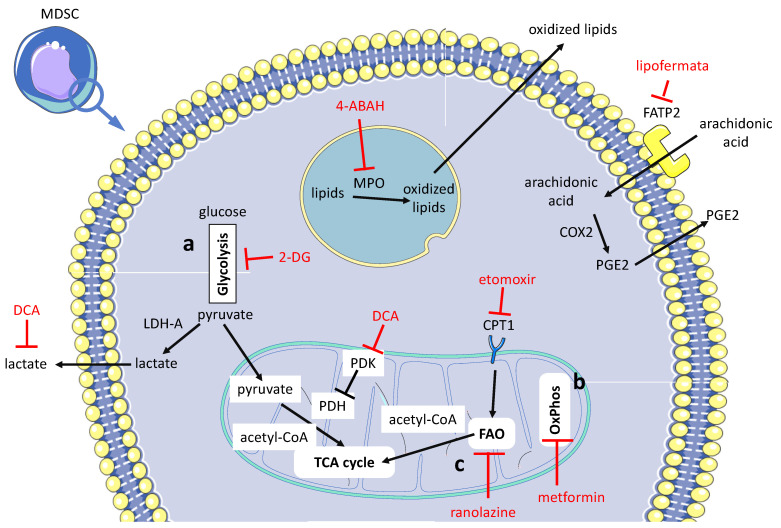
**Pharmacological treatments aimed at counteracting in the TME the immunosuppressive activity of granulocytic MDSCs’ three main targets**. (**a**) Dampening glycolysis [107,108], (**b**) restraining OXPHOS [109] and (**c**) hindering/modulating lipid metabolism through FAO inhibition [110], inhibition of arachidonic acid uptake [111] and inhibition of lipid peroxidation [112]. The cell targets of drug treatment are: PMN-MDSCs [109,111,112]; both PMN-MDSCs and M-MDSCs [108,110], and the whole MDSC cell population [107,113]. Therapeutic interventions are outlined in red. For explanation of acronyms and further details, refer to the text. Lipid peroxidation by MPO occurs in lysosomes.

To our knowledge, no article has been published yet on the drug rewiring of neutrophil metabolism to aid anti-tumor immunity and improve immunotherapy. Below, we summarize several preclinical studies carried out on murine MDSCs, in which two main subpopulations have been shown to exist: PMN-MDSCs (CD11b^+^Ly6G^+^Ly6C^lo^) and M-MDSCs (CD11b^+^Ly6G^lo^Ly6C^hi^) [5,23]. While in the case of NK cells, the goal is their reprogramming of metabolism to promote effector functions, in the case of MDSCs, the goal is generally the reverse, that is, their depletion from the TME or even inhibition of differentiation from bone marrow precursors [5,21,23]. For NK cells, pharmacological strategies aim at restoring glycolysis, and thus promoting effector function against tumors; the opposite mechanism is crucial for MDSCs. Indeed, MDSCs depletion from TME or even differentiation inhibition is under investigation in preclinical studies. It follows the description of a few preclinical studies of pharmacological modulation of metabolism in MDSCs aimed at supporting anti-tumor therapies, as shown in Figure 3.

Tumor-associated MDSCs exhibit a high glycolytic rate. This metabolic state, via the antioxidant activity of glycolytic intermediates, prevents excess ROS production, protecting MDSCs from apoptosis. Blockade of glycolysis by treatment with the hexokinase inhibitor 2-deoxy-D-glucose (2-DG) produces decreased numbers of MDSC in TME and suppresses tumor growth in the orthotopic 4T1 murine model of breast cancer [108]. Targeting aerobic glycolysis by dichloroacetate (DCA) (a pyruvate dehydrogenase kinase (PDK) inhibitor) improves oncolytic viro-immunotherapy in cancer-bearing mice. Within the TME, DCA treatment reduces lactate release, STAT3 activation, IDO1 over-expression and MDSC infiltration, leading to enhanced anti-tumor immune response and prolonged survival of mice. DCA treatment decreases infiltrating MDSCs by inhibiting glycolysis in both MDSCs, and in other cells in the TME, it counteracts lactic acidosis, which in turn promotes MDSC differentiation [107]. Metformin redirects the metabolism of PMN-MDSCs, reducing OXPHOS and enhancing glycolysis, thereby pushing the microenvironment to a state that inhibits the growth of certain tumors [109].

Hypoxia is a key feature of TME and correlates to poor prognosis; moreover, hypoxic tumor areas recruit immunosuppressive MDSCs in humans and in mice [114]. To alleviate hypoxia in the TME, silica nanoparticles co-loaded with the photosensitizer IR-780, metformin and CeO_2_ were used in mice for the therapy of subcutaneous tumors. Thanks to CeO_2_ etching with endogenous H_2_O_2_, this nanodevice can selectively release its molecular cargo, producing an overall increase in local O_2_. Furthermore, in this model, metformin saves intracellular O_2_ through the inhibition of mitochondrial respiration, thus working as an “O_2_ economizer”. As a result of decreased hypoxia, MDSCs are locally reduced, and anti-tumor immunity is boosted upon laser irradiation [113].

Studies on glucose metabolism draw the opposite conclusion about the role of glycolysis in regulating MDSCs’ behavior. Some of the reported studies indicate that glycolysis is crucial for MDSCs’ immunosuppressive functions, while others highlight that glycolysis enhancement causes MDSCs’ function inhibition. In fact, in some studies, it is stated that an inhibition of glycolysis corresponds to a decrease in MDSCs [108], while in others, it is said that metformin, a OXPHOS inhibitor and indirect enhancer of the glycolytic pathway, produces an inhibition of MDSCs [109,113].

Mice receiving a PUFA-enriched diet have been shown to foster the differentiation of MDSCs from bone marrow precursors and to potentiate the suppressive activity of these cells [115]. In addition, the microenvironment in different types of neoplasia is enriched in lipids [115]. Accordingly, diverse recent studies have shown that, compared to glucose metabolism, lipid metabolism is probably a more promising therapeutic target to counteract MDSCs.

In TME, the uptake of lipids, mediated by the scavenger receptor CD36, promotes in tumor-associated MDSCs the switch from glycolysis to FAO [110]. FAO inhibition by etomoxir or by ranolazine dampens immunosuppressive functions in MDSCs, which results in delayed tumor growth and improved immunotherapy [110].

In PMN-MDSCs, the fatty acid transport protein 2 (FATP2) mediates the uptake of arachidonic acid, a substrate for the synthesis of the strongly immunosuppressive PGE_2_. The FATP2 inhibitor lipofermata reverses the suppressive potential of PMN-MDSCs, supporting cancer immunotherapy [111]. Through the transfer of oxidized lipids to dendritic cells, PMN-MDSCs negatively affect the cross-presentation function in these cells. In PMN-MDSCs, oxidized lipids are synthetized by lysosome-located myeloperoxidase (MPO). In vivo treatment with MPO inhibitor 4-ABAH in combination with checkpoint blockade reduces tumor growth [112].

## 6. Considerations for Targeting Tumor Metabolism: A Focus on Neutrophils, PMN-MDSCs and NK Cells

This section examines a few questions arising from the studies just described. The considerations presented address relevant issues to be envisaged for approaches efficiently targeting tumor metabolism.

### 6.1. Coexistence of Different Metabolic Pathways in the Same Cell

To interpret the evidence presented above, it is important to consider that cells can simultaneously exploit different pathways for energy metabolism, even if there is often one that prevails [116]. This fact explains why the inhibition of distinct metabolic pathways, which seem to be regulated in an antagonistic manner, can result in the same outcome. The inhibition of immunosuppressive activity in MDSCs can be proposed as an example.

### 6.2. Cellular Heterogeneity in the TME

The TME is characterized by extreme heterogenicity in its cell compositions. For this reason, it is often difficult to establish the targets of drug treatments. Moreover, drug therapies aimed at metabolic rewiring may favor prognosis on one hand but produce damage on the other. In other words, they can counteract the immunosuppressive environment while promoting the proliferation of cancer cells. In the case of NK cells, this problem can be solved at the root by treating/modifying the cells ex vivo for later use in ACT procedures.

It could be speculated that different metabolic profiles exist in diverse MDSC subsets. Therefore, there are still not sufficient studies to support the feasibility of targeting a specific MDSC subset employing a defined metabolic profile (PMN-MDSCs vs. M-MDSCs) at the current stage. Another relevant point to be considered, for therapeutic interventions, is the short half-life of MDSCs. Therefore, the most promising therapeutic strategy could be to inhibit differentiation from bone marrow precursors or inhibit recruitment to the TME, rather than pursuing the goal of simple depletion in the TME [21,22]. In addition, we must also consider that MDSCs are immature cells, and the goal could be to push them to differentiate towards anti-tumor or at least non-relevant cell types [117].

### 6.3. Pathophysiological MDSCs

It is possible that the puzzling evidence regarding MDSC metabolism stems in part from the fact that pathological aspects of this heterogeneous cell population are well investigated, while much less is known about their likely role in maintaining tissue homeostasis [118]. For this reason, it is problematic to define metabolic reprogramming in these cells; indeed, it is not known whether these cells originate in the bone marrow or in peripheral tissues following conversion from physiological cell counterparts.

### 6.4. Immunosuppressive Activity of MDSCs on T Cell Responses

In most of the preclinical models discussed [119,120], it is shown that the inhibitory activity of MDSCs is specifically directed against T responses and probably NK cell responses, although it has generally not been investigated. In fact, the therapeutic effect may be thwarted in T-cell-depleted mice.

### 6.5. Modulation of Lipid Metabolism, Similar Outcomes in NK Cells and MDSCs

A lipid-rich tumor microenvironment, but also a state of obesity preceding tumorigenesis, simultaneously contribute to hypo-functional NK and hyper-active MDSCs [22,104]. Thus, acting on lipid metabolism could simultaneously favor anti-tumor NK and inhibit pro-tumor MDSCs, contributing to the success of cancer chemotherapy and immunotherapy.

## 7. Conclusions

Most of the original articles discussed in this review date back to the last three years, indicating that the pathophysiological contexts studied concern rapidly evolving areas of investigation. Moreover, even when the involvement of neutrophils or PMN-MDSCs and NK cells is well documented, the mechanistic aspects have often not been investigated. In general, it is likely that the involvement of neutrophils, PMN-MDSCs or NK cells in cancer metabolism is much more widespread than has been reported so far. Finally, what is more, functional interactions between NK cells and neutrophils in the pathophysiological settings discussed in this article are probably still largely to be discovered. Some support for these studies may come from the fact that neutrophil-like or NK-like leukocyte populations have already been shown to be implicated in cancer metabolism effects on the TME.

Among the promising areas for future research within the topic, there are several approaches that could be envisaged, including: (a) investigation of the mechanisms by which host microbiota that colonize the gut, but also other body surfaces could exert a significant influence on metabolism in neutrophils, MDSCs and NK cells; (b) investigation of the metabolic landscape in regulatory, cytokine-producing, and pro-angiogenic NK cell subsets. In physiological conditions, these cells are found in high amounts in decidua during pregnancy. Moreover, actively pro-tumor NK cells mainly belong to the category of regulatory NK cells; (c) a better characterization of physiological MDSCs and their metabolic features. These cells may exert a beneficial role at sites of chronic inflammation that may eventually progress into pre-cancerous lesions. A possible example has been reported in chronic alcoholic hepatic damage [121]; (d) as thoroughly discussed in the text, a deeper insight into lipid metabolism in NK, neutrophils, MDSCs and even T cells may be of the utmost importance for the design of novel therapeutic approaches to cancer immunotherapy.

Moreover, an intriguing topic in this field of study is the potential “symbiotic” interactions within the TME. For example, it is tempting to speculate that, whereas with some subsets of leukocytes (M1, anti-tumor T cells effectors, anti-tumor NKs), cancer cells have predominantly antagonistic metabolic relationships, with other subsets (Treg, MSDCs, M2, N2) cancer cells can establish types of metabolic cooperation. Currently, to the best of our knowledge, there is little direct experimental support for this relevant aspect. The hypothesis originates from the following preliminary evidence, suggesting antagonistic metabolic interactions between tumor cells and anti-tumor immune effectors: (a) T regs, M2 and MDSCs, i.e., potentially pro-tumor subsets, rely substantially on FAO and thus on fatty acid availability. In contrast, effector T cells, M1 and NK cells rely primarily on aerobic glycolysis and thus glucose availability [122]; (b) in TME, glucose concentration is frequently in limiting amounts [123], whereas the situation for fatty acids and other lipids is substantially different. These nutrients are not in limiting amounts, but instead are often abundant [124].

The cooperative metabolic interaction hypothesis is supported by the examples discussed in Section 4.6, where neutrophils with a pro-tumor phenotype implement metabolism in neoplastic cells.

Finally, in a broader perspective of evolutionary biology, cancer cells co-opt pre-existing physiological mechanisms and exploit and hijack them to increase their replicative fitness. This evolutionary dynamic is evident in many areas of oncology and thus holds true also for the energy metabolism of cancer. The ability of cancer to reuse pre-existing physiological mechanisms can be considered a special case of the evolutionary tinkering dynamics advanced in 1977 by Nobel laureate Francois Jacob [125].

## Data Availability

Schematic figures were created with images adapted from Smart Servier Medical Art (http://www.servier.fr/servier-medical-art).

## Abbreviations

ADCC	Antibody-dependent cellular cytotoxicity
ATC	Adoptive cell transfer
ATGL	Adipose triglyceride lipase
ATP	Adenosine triphosphate
CAR	Chimeric antigen receptors
CeO_2_	Cesium oxide
CPT1	Carnitine palmitoyl transferase 1
DCA	Dichloroacetate
dNK	Decidual natural killer
Drp1	Dynamin-related protein1
ECM	Extracellular matrix
FAO	Fatty acid oxidation
FATP2	Fatty acid transport protein 2
FBP-1	Fructose-1,6-bisphosphatase 1
GM-CSF	Granulocyte–macrophage colony-stimulating factor
IFNγ	Interferon gamma
IL	Interleukin
IL-15R	Interleukin-15 receptor
iLDNs	Immature low-density neutrophils
iPSC	Induced pluripotent stem cells
LDH-A	Lactate deydrogenase-A
M1/2	M1/2 macrophage
MDSCs	Myeloid-derived suppressor cells
MHC	Major histocompatibility complex
MPO	Myeloperoxidase
mTOR	Mammalian target of rapamycin
N1/2	N1/2 neutrophils
NADPH	Nicotinamide adenine dinucleotide phosphate hydrogen
NETs	Neutrophil extracellular traps
NK	Natural killer
OXPHO	Oxidative phosphorylation
P2RX1	Purinergic receptor P2X 1
PaD4-KO	Peptidyl arginine deiminase 4 knock out
PD-1	Programmed cell death protein 1
PDK	Pyruvate dehydrogenase kinase
PDL-1	Programmed death ligand 1
PGC-1a	Peroxisome proliferator-activated receptor gamma coactivator 1 alpha
PGE_2_	Prostaglandin E_2_
PPAR-γ	Peroxisome proliferator-activated receptor gamma
PUFA	Poly-unsaturated fatty acids
ROS	Reactive oxygen species
SCF	Stem cell factor
SREBP	Sterol regulatory element-binding protein
STAT3	Signal transducer and activator of transcription 3
TAM	Tumor-associated macrophages
TAN	Tumor-associated neutrophil
TCA	Tricarboxylic acid
TGFβ	Transforming growth factor beta
Tigit	T cell immunoreceptor with Ig and ITIM domains
TIM3	T-cell immunoglobulin and mucin domain-3
TLR4	Toll-like receptor 4
TME	Tumor microenvironment
TNFα	Tumor necrosis factor alpha
TORC1/2	Target of rapamycin complex1/2
VEGF	Vascular endothelial growth factor
